# Evaluation of the isoniazid preventive therapy (IPT) program in Shurugwi District, Midlands Province, Zimbabwe, January 2013 to August 2014

**DOI:** 10.1186/s13104-015-1451-y

**Published:** 2015-09-25

**Authors:** Annamercy Makoni, Milton Chemhuru, Mufuta Tshimanga, Notion Tafara Gombe, More Mungati, Donewell Bangure

**Affiliations:** Department of Community Medicine, University of Zimbabwe, Office 3-66 Kaguvi Building, Cnr 4th/Central Avenue, Harare, Zimbabwe; Ministry of Health and Child Care, Harare, Zimbabwe

**Keywords:** IPT program evaluation, Shurugwi District, Midlands Province

## Abstract

**Background:**

Midlands Province started implementing the Isoniazid (INH) preventive therapy (IPT) program in January 2013. Shurugwi and Gokwe North were the piloting district hospitals. In May 2014, four more districts hospitals (Gokwe South, Gweru, Kwekwe and Zvishavane) started implementing IPT. Shurugwi District decentralized the program to its rural health facilities in January 2014. A review of the Shurugwi IPT program, 2013 data, indicated that the majority of eligible clients were not started on IPT. None out of the 400 eligible clients were started on IPT in November against the 100 % target according to the World Health Organization and the National Tuberculosis (TB) Program. We conducted a study to evaluate the IPT program in Shurugwi District from January 2013 to August 2014.

**Methods:**

The logical framework approach was used to evaluate inputs, processes, outputs and outcomes of the IPT program. An interviewer administered questionnaire was used to collect data from key informants. Checklists were used to collect data from IPT program records.

**Results:**

Sixteen health facilities were implementing IPT in Shurugwi District. All the facilities had TB screening tools and three did not have TB screening algorithms. The district experienced medicine stock outs in 2013. One formal training at district level and on job trainings in implementing health facilities were done. From January 2013 to August 2014, Shurugwi District screened 6794 antiretroviral (ART) clients for TB. Out of those screened, 5255 were eligible for IPT and 2831 (54 %) were started on IPT. A total of 700 clients had completed the IPT 6 month’s course by August 2014. The dropout rate due to INH toxicity and TB was 0.6 % (n = 18) and 0.3 % (n = 8) respectively. Fifty-three advocacy and community sensitization meetings were done. The program had no Information Education and Communication (IEC) materials.

**Conclusion:**

The IPT program in Shurugwi District achieved half its target. This could be due to inadequate formally trained staff, lack of IEC materials, inadequate advocacy and community sensitization, non-availability of the INH 300 mg single dose and inadequate INH 100 mg dose tablets in 2013. To improve the IPT program, there is need for routine advocacy, communication and social mobilization.

## Background

Isoniazid Preventive Therapy (IPT) is the provision of isoniazid (INH) to people at high risk of developing active tuberculosis (TB) [1]. It is a form of secondary prevention involving the provision of INH to prevent progression of latent TB to active TB. People with the Human Immunodeficiency Virus (HIV) are 20–37 times more likely to develop active TB from latent TB than those without HIV [1]. HIV infection is the strongest risk factor for developing tuberculosis and has fuelled its resurgence, especially in sub-Saharan Africa [1]. In 2010, there were an estimated 1.1 million incident cases of tuberculosis among the 34 million people living with HIV worldwide [2]. It is estimated that between 30 and 50 % of HIV positive individuals die of TB, hence the need to prevent TB infection in HIV infected individuals through administration of IPT for 6 months [2]. Studies have shown that IPT for up to 6 months may prevent TB infection in HIV positive clients [2]. Providing isoniazid to prevent TB among people living with HIV is a proven and internationally recommended strategy that has been effectively implemented in low resource settings [1].

The World Health Organization (WHO) recommends IPT as part of the three I’s for HIV/TB collaboration. These include, intensified TB case finding, INH preventive therapy and Infection control for TB in health care and congregate settings [1]. The current recommended dose of IPT for adults is 300 mg of INH per day for 6 months, with 36 months conditionally recommended in areas of high TB prevalence and transmission [1].

Adults and adolescents living with HIV should be screened with the WHO symptom checklist for TB, to rule out active TB infection. Those reporting one or more symptoms may have active TB and should be further evaluated and then treated for TB [3].

WHO has recently revised the guidelines on IPT that recommends the use of a simplified screening algorithm that relies on the absence of all four clinical symptoms (current cough, night sweats, fever, and weight loss) to identify people living with HIV (PLHIV) who have less likelihood of active TB disease and hence eligible for IPT [4]. This simplified symptom-based algorithm should be used for all adults living with HIV, including pregnant women, antiretroviral therapy (ART) clients and those who have successfully completed TB treatment [4].

According to WHO, before initiating a service to provide routine IPT to PLHIV, some prerequisites should be in place. These are a strong linkage between HIV care and TB control services, adequate capacity for HIV counseling, which should include information, education and counseling about TB, health care staff well trained in pre-treatment screening to exclude active pulmonary and extra pulmonary TB, resources available for close monitoring, supervision and evaluation of IPT outcomes, TB control programme with high adherence, high cure rates (≥70 %), case detection (≥70 %) and combined default/failure rates of less than 10 % [3].

Globally, almost a quarter of a million PLHIV, that is 242,847, were reported to have received IPT between the period 2002 to 2009, showing a 19-fold increase over the period [3]. Despite the barriers and low uptake of IPT globally, IPT has been successfully implemented in several settings. [3].

In sub-Saharan Africa, where the emergence of HIV has caused dramatic increases in tuberculosis (TB) case notifications, IPT for HIV positive individuals was evaluated as a potential community-wide strategy for improving TB [2]. Zimbabwe continues to experience a major HIV driven TB epidemic with co-infection rates of 82 % [5]. Considerable progress has been made towards addressing the 12 point WHO collaborative TB/HIV activities. As of 2011, 92 % of all TB patients notified during the year had an HIV test result, 85 % of the HIV positive TB patients received cotrimoxazole and 71 % received ART [5]. Progress on implementation of the 3I’s has been very slow especially Isoniazid Preventive Therapy (IPT). Implementation of the intensified TB case finding (ICF) and isoniazid preventive therapy (IPT) initiative started in December 2012 with a feasibility phase being commissioned in 10 selected sites across 5 provinces [5].

By the end of June 2014, the number of sites implementing ICF/IPT had expanded to 46 in total with each of the 10 provinces in the country having at least two (2) to six (6) district level institutions [5]. The 46 health facilities included 27 district hospitals, 1 provincial hospital and 2 central hospitals and a few clinics in Bulawayo City and Harare City. From January 2013 to May 2014, Zimbabwe was reported to have screened 425 190 HIV positive clients for TB. Out of the screened clients, 206,121 (48 %) were eligible for IPT and only 47,791 (23 %) of these were started on IPT. A total of 19,034 HIV positive clients were reported to have completed IPT [5].

Midlands Province started implementing IPT program in January 2013. Shurugwi and Gokwe North were the piloting district hospitals [6]. In May 2014, four more districts hospitals (Gokwe South, Gweru, Kwekwe, and Zvishavane) in the province started implementing the IPT program. Shurugwi District hospital started decentralizing the program to its rural health facilities in January 2014 [6].

A review of the Shurugwi IPT program, 2013 data, indicated that the majority of clients, eligible for IPT were not started on IPT. In 2013, three out 62 (5 %), zero out of 400 (0 %) and 4 out of 400 (1 %) of the eligible HIV positive clients were started in August, September and November respectively [7]. The target for IPT initiation is 100 % according to WHO and the National TB Program (NTP). All eligible clients are expected to be started on IPT. In December 2013, the National TB Programme with close consultation with the Midlands Provincial Medical Director (PMD) and the District Health Executive (DHE) for Shurugwi District recommended the decentralization of the IPT program to rural health facilities in Shurugwi District. During the period under study, there were 16 health facilities in the district that were either ART initiating or follow up sites providing IPT. The IPT program was never evaluated.

## Methods

A descriptive study was conducted in Shurugwi District. The logical framework approach was used to evaluate inputs, processes, outputs and outcomes of the IPT program in Shurugwi District. Nurses in Charge (NIC) from the 15 health facilities, Opportunistic infections (OI)/ART Sister in Charge (SIC) from the district hospital, the District Medical Officer (DMO), TB coordinator, District Nursing Officer (DNO), Matron and Pharmacist were purposively recruited into the study as key informants. An interviewer administered questionnaire was used to collect data from key informants on resource availability, IPT processes being implemented both at district, health facility and community level, IPT program achievements and challenge. Checklists were used to collect data from OI/ART records, TB records and IPT registers.

Ethical approval was obtained from the Provincial Medical Director (PMD) for Midlands Province, Health Studies Office (HSO), and DMO for Shurugwi District. Confidentiality was assured and maintained throughout the study. Participants were told that there were no monetary benefits for participating and that participation was voluntary. Informed written consent was obtained from each participant.

## Results

### Demographic characteristics of key informants, Shurugwi District, Midlands Province, 2014

Out of the 21 study participants, the majority (71.4 %) were Nurses in Charge of the 15 health facilities (excluding the district hospital) offering IPT in Shurugwi District. The median years in service was 8 years (Q1 = 3; Q3 = 25) (Table 1).

**Table 1:** Demographic characteristics of key informants, Shurugwi District, Midlands Province, Zimbabwe, 2014

Variable	Frequency n = 21	Percentage
Sex		
Male	6	29
Female	15	71
Designation		
Nurses in charge	15/15	71.4
District Medical Officer	1/1	4.8
OI/ART Sister in Charge	1/1	4.8
District Nursing Officer	1/1	4.8
Matron	1/1	4.8
Pharmacist	1/1	4.8
TB coordinator	1/1	4.8
Years in service	Median: 8 (Q_1_ = 3; Q_3_ = 25)	

### Inputs injected into the IPT program, Shurugwi District, Midlands Province, January 2013 to August 2014

The IPT program started in January 2013 at the district hospital and was decentralized to 15 other health facilities in January 2014. The number of health workers for the IPT program was 77 % (77 out of 100) the required. In 2013 the district hospital had both IPT guidelines and TB screening tools. In 2014 all the 16 health facilities had TB screening tools and only 3 did not have IPT guidelines. The district never produced or received Information, Education and Communication (IEC) materials on IPT. All the 175 village health workers (VHW) were sensitized on IPT in 2013 (Table 2).

**Table 2:** Inputs used to running the IPT Program, Shurugwi District, Midlands Province, Zimbabwe, 2013/2014

Item	Jan–Dec 2013 (1 site)	Target	Jan–Aug 2014 (16 sites)	Target
Health workers involved in IPT	14	14	77	100
TB/IPT screening algorithms	2	2	17	20
TB screening tools	2	2	31	31
INH available for the period	224 boxes	840 boxes	3192 boxes	8200 boxes
Pyridoxine available for the period	75 bottles	288 bottles	1384 bottles	2952 bottles
Vehicle	1	1	1	1
Motor cycles	1	1	6	11
IEC materials (pamphlets)	0	2000	0	20,000

### Processes involved in running the IPT program in Shurugwi Districts, Midlands Province, 2013/2014

One formal training was conducted by the district and on job trainings were done in all health facilities in 2013 and 2014. Advocacy and community sensitizations were mainly done in 2013 and were inadequately done in 2014.

### Outputs of the IPT Program, Shurugwi District, Midlands Province, Jan 2013–Aug 2014

The district decentralized the IPT program to 15 health facilities in January 2014. A review of clinic records suggested that from January 2013 to August 2014, Shurugwi district had screened 6794 (92 %) ART clients for TB. Out of those screened, 5255 (77 %) were eligible for IPT and 2831 (54 %) were started on IPT. About 10 (63 %) of the health facilities conducted a total of 53 community sensitizations on IPT. However, the program had no IEC materials to compliment community sensitizations (Table 3).

**Table 3:** Outputs of the IPT program in Shurugwi District, January 2013 to August 2014

Indicator	Jan–Dec 2013 (1 site)	Target	2014 Jan–Aug (16 sites)	Target	Jan 2013–Aug 2014 (16 sites)
OI clinic burden (cumulative for repeat visits)	10,347	10,347	19,381	19,381	29,728
Number of ART clients	6559	–	7385	–	7385
Number of ART clients screened for TB	6559	–	6449	–	6794
Number eligible for IPT	1797	1797	3458	3458	5255
Number started on IPT	933	1797	1898	3458	2831
Proportion of eligible clients started on IPT	52 %	100	55 %	100	54 %
Number of dropouts due to toxicity	1	0	17	0	18
Number of clients who developed TB during IPT	2	0	6	0	8
Number completed IPT	335	338	365	388	700
Number of Health facilities offering IPT	1	1	16	11	16
Number of HW trained on IPT (formal training)	41	77	0	36	41
Number of VHWs sensitized on IPT	175	175	0	175	175
Number of IEC materials on IPT distributed	0	2000	0	20,000	0
Number of IPT advocacy and sensitization meetings	4	12	49	100	53
Number of facilities doing advocacy and community sensitizations	1	1	10	16	11

### Outcomes of the IPT Program, Shurugwi District, Midlands Province, January 2013 to August 2014

From January 2013 to August 2014, Shurugwi District started 54 % of the ART clients on IPT. A total of 700 clients completed the INH 6 months course. Six (0.6 %) of the clients stopped IPT due to INH toxicity and eight (0.3 %) clients developed TB during IPT.

## Discussion

IEC materials provide key messages to educate communities on the benefits of IPT, thereby marketing the program and clearing myths and misconceptions about IPT. Shurugwi District had no IEC materials on IPT. The National TB Program did not produce IEC materials to educate the public. Indumathi et al. found that program uptake can be enhanced by adequate education at the start of the therapy and during subsequent follow ups [7]. This implies that, community education through the distribution of posters, fliers and pamphlets is necessary to improve awareness and program uptake.

Advocacy and community sensitization are strategies that are implemented to enhance community participation and involvement of local leadership. If communities are involved and gain the ownership of the program, uptake improves. Advocacy and community sensitization for program success was not well capacitated by the district hence low program uptake. The district conducted advocacy and community sensitizations for the IPT program mainly when the program started in 2013. We concluded that there was need to continue advocating for the program through adequate involvement of the local community leadership and the community at large in program implementation. Similar findings were reported by Durovni et al. where he recommended advocacy efforts for effective scale up of the program [8].

The target for IPT initiation is 100 % according to the World Health Organization and the National TB Program. All eligible clients are expected to be started on IPT. In Shurugwi District, half of the eligible clients were started on IPT, however this was more than double the national picture where almost a quarter of the eligible clients were started on IPT from January 2013 to May 2014. INH stock outs were experienced in 2013 when the program started leading to failure to start some clients on IPT in that same year. In Shurugwi District it was noted that some clients declined IPT due to fear of pill burden as they were already on ART and Cotrimoxazole, making a minimum of 10 tablets per day for those who accepted IPT.

All clients started on IPT are expected to complete the 6 months course. Treatment completion was high mainly because a small proportion stopped due to development of INH toxicity and TB during IPT. Completion of the IPT course implies successful control of TB among ART clients for a period of 2–3 years after IPT. Similar findings were reported by, Durovni et al. in 2010 who recorded a high adherence and completion rate among ART clients [8].

Screening algorithms provide important information on eligibility criteria. Lack of IPT screening algorithms may result in poor program implementation. The majority of the health facilities offering IPT in this study had TB screening algorithms. Granich et al., in 2010, found out that lack of standard operating procedures (SOPs), guidelines and screening algorithms contributes to the low availability and poor uptake of IPT [9]. WHO highlighted that the lack of guidelines was a barrier for IPT provision rather than patient related factors [10]. However, all health facilities were using TB screening tools for all clients, either HIV positive or not. This implies intensified case finding and is part of the 3Is for HIV/TB collaboration recommended by WHO [10].

In this study, we found out that the district hospital managed to decentralize the program to 15 other health facilities which is a positive achievement. However, challenges faced were shortage of staff in some clinics. Health workers were inadequate and overwhelmed because of many other competing program activities that call for their daily attention for example Provider Initiated Testing and Counseling (PITC), TB screening and treatment, (OI)/ART, Expanded Program on Immunization (EPI), Child growth monitoring, Prevention of Mother to Child Transmission of HIV (PMTCT) and deliveries. With adequate staff, IPT processes would be adequately implemented.

It was observed that the IPT program was highly effective at screening TB among ART clients, this is intensified case finding. The majority of ART clients were screened for TB at every review visit. This implies early detection and management of TB, hence reduced transmission to others. The key informants highlighted that It was also during clinic visits that clients were assessed for adherence to treatment through pill count.

Velle et al. found out that the most common reason for failure to adhere to and complete treatment was not visiting the health facility frequently [11]. In this study treatment completion was high, a few clients developed INH toxicity and TB during IPT. Half of the key informants interviewed highlighted that they were using a treatment buddie (close family member) to monitor adherence to treatment. Nolan et al. found out that IPT clients enrolled on community based directly observed preventive therapy completed their treatment; hence a close monitoring of clients promotes treatment completion [12].

## Conclusion

The IPT program in Shurugwi District achieved half its target. This could be due to inadequate formally trained staff, lack of IEC materials, inadequate advocacy and community sensitization, non-availability of the INH 300 mg single dose and inadequate INH 100 mg dose tablets in 2013. The district should improve Advocacy, Communication and Social Mobilization (ACSM) for the IPT program.

## Public health actions taken

As a result of this study, we gave health education on IPT to 15 ART clients, sourced and distributed A4 size TB/IPT screening algorithms to the clinics in need. We corrected some anomalies noted in IPT implementation where some clinics were not prescribing Pyridoxine together with INH to reduce the risk of developing side effects.

## Recommendations

The study recommended the DMO to train health workers who were not formally trained on IPT. The District Health Promotion Officer to produce and distribute IEC materials on IPT for educating health workers, clients and the community at large and to conduct advocacy, communication and social mobilization (ACSM) for IPT. Nat Pharm to provide the 300 mg single dose of INH for the IPT programme in order to reduce pill burden for clients.

## Abbreviations

ART: antiretroviral therapy
CDC: center for disease control and prevention
DHE: District Health Executive
DMO: District Medical Officer
DNO: District Nursing Officer
EPI: Expanded Program on Immunization
HIV: human immunodeficiency virus
ICF: intensified case finding
IEC: information, education and communication
INH: isoniazid
IPT: isoniazid preventive therapy
SIC: Sister in Charge
NTP: National TB Program
OI: opportunistic infections
PITC: provider initiated testing and counseling
PLWHIV: people living with HIV
PMD: Provincial Medical Director
PMTCT: prevention of mother to child transmission of HIV
SOPs: standard operating procedures
TB: tuberculosis
VHW: village health workers
WHO: World Health Organization
